# Healing in the colourful HELIUS experience

**DOI:** 10.1007/s12471-018-1108-2

**Published:** 2018-04-11

**Authors:** R. Delewi, J. J. Piek

**Affiliations:** Department of Cardiology, Academic Medical Center, University of Amsterdam, Amsterdam, The Netherlands

Over the past decades, clinical guidelines have become an increasingly important aspect of clinical practice and every day international guidelines influence the clinical decisions we make at the patient’s bedside. We define evidence-based medicine as a conscientious, explicit and judicious use of current best evidence in the decision-making process in the care of individual patients [1].

As doctors we realise that guidelines should only be used as a starting point in our clinical decision-making. In daily practice, we must incorporate the cultural, social and economic contexts of the patient for the treatment to be a success. Patient characteristics appear to exert influence: for instance, co-morbidity in a patient appears to reduce the chance that guidelines are followed [2]. It has been postulated that professionals assume that guidelines are based on a general clinical picture and that they are insufficiently tailored to the often complex care requirements of patients with different backgrounds [3].

In the prevention of coronary heart disease and cardiovascular disease we focus on reducing the overall absolute risk of disease rather than on managing individual risk factors. Here, cultural, social and economic contexts are now more important than ever. Estimating the total cardiovascular risk is a crucial part of our clinical practice with the obvious statement being that those at highest risk from a cardiovascular disease event gain most from preventive measures. This can only be achieved with a holistic approach.

Meanwhile, populations across the world are becoming increasingly ethnically diverse due to globalisation. Health risks seem to differ between ethnic groups. Most large population-based cohort studies have excluded ethnic minority populations [4]. The Netherlands in that sense offers a unique study cohort. According to Eurostat, in 2010 there were 1.8 million foreign-born residents in the Netherlands, corresponding to 11.1% of the total population. Of these, 1.4 million (8.5%) were born outside the EU. This will probably increase to 20% by 2060 [5].

In this issue, Perini et al. describe an elegant observation from the Dutch HELIUS (HEalthy LIfe in an Urban Setting) study that was designed to unravel potential mechanisms underlying the impact of ethnicity on communicable and non-communicable diseases [6]. The authors estimated the risk of fatal cardiovascular disease and the risk of fatal plus nonfatal cardiovascular disease using the SCORE algorithm in native Dutch, South Asian Surinamese, African Surinamese, Ghanaian, Turkish and Moroccan citizens. In the article they demonstrate that ethnic minority groups are at a greater estimated cardiovascular risk. Relative to the native Dutch, South Asian Surinamese showed a higher risk of fatal CVD. African Surinamese and Turkish men showed a higher risk of a combination of fatal and non-fatal cardiovascular disease [7].

We applaud the researchers for using the HELIUS study to get more data on the colourful practice that we face every day. It is a beautiful observation to describe different cardiovascular risk profiles per ethnicity. If we want to take this observation to the next level, however, more questions arise. Are these observations due to genetics or due to lifestyle? Did the participants have the same access to the health care system? What is their level of education and participation in society? It is only when we find the answers to these questions that we can really make a difference.

After all, the prevention of cardiovascular disease relies on the reduction of the overall absolute risk of disease rather than management of individual risk factors. It appears that there is a lot to gain in specific ethnic minorities. The next step should be that we determine how we can improve prevention of cardiovascular disease in these groups. We certainly will need to individualise our prevention programs for ethnic-specific prevention strategies.

But even a categorisation based on the ethnicity of patients is an oversimplification of the truth. Every day, we are faced with different views on health, lifestyle and the quality of life in general, regardless of background, with no guidelines or SCORE calculators to help us incorporate these different dimensions in clinical decision-making. So, is it possible to create international standards that will fit the cultural, social, and economical contexts of each unique patient? Fortunately, we as doctors can still be of added value in the individual patient care in daily practice.
